# The potential roles of retinoids in combating drug resistance in cancer: implications of ATP-binding cassette (ABC) transporters

**DOI:** 10.1098/rsob.220001

**Published:** 2022-06-01

**Authors:** Mohamed R. Abdelaal, Hesham Haffez

**Affiliations:** ^1^ Biochemistry and Molecular Biology Department, Faculty of Pharmacy, Helwan University, Cairo 11795, Egypt; ^2^ Centre of Scientific Excellence ‘Helwan Structural Biology Research (HSBR)’, Helwan University, Cairo 11795, Egypt

**Keywords:** MDR, ABC transporter, P-glycoprotein, BCRP, MRP-1, retinoids

## Abstract

Multidrug resistance (MDR) means that tumour cells become unresponsive during or after the course of treatment to one or more of chemotherapeutic drugs. Chemotherapeutic resistance critically limits the treatment outcomes and remains a key challenge for clinicians. The alternation in intracellular drug concentration through the modulation of its transport across the plasma membrane is the major cause for MDR and is adopted by various mediators, including ATP-requiring enzymes (ATPases). Among these ATPases, ABC transporters have been extensively studied, and found to be highly implicated in tumorigenesis and MDR. The present review sheds light on the documented effects of retinoids on ABC enzymes to understand their mechanism in combating cancer cell resistance. This would open the gate to test the mechanism and applicability of different new synthetic retinoids in literature and market as modulators of ATP-dependent efflux pumping activity, and promote their applicability in diminishing anti-cancer drug resistance.

## Graphical abstract

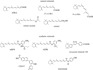

## 1. Introduction

Multidrug resistance (MDR) occurs when cancer cells become progressively unresponsive to anti-cancer drugs independently of their structures and/or mechanisms of action [1]. MDR might arise due to alteration in drug target molecules, interrupted access to target cells, genetic responses, enhanced DNA repair mechanisms, counteracting growth factors, metabolic effects or altered transport of the chemotherapeutic agent across the plasma membrane [1–4]. The latter mechanism is mediated by a wide range of ATP-requiring enzymes (ATPases). ATPase family members are indispensable enzymes for both normal and cancer cells [5]. They are widely distributed within cells and differ considerably in structures and biological activities. They share the ability to hydrolyse the phosphate *γ*–*β* bond of ATP to release free energy that is harnessed subsequently by the enzyme to perform its biological functions [6]. ATPase superfamily comprises ATP-binding cassette (ABC) transporters, P-type ATPases, V-type ATPases, kinesins, helicases, heat-shock proteins as well as ATPases associated with different cellular activities (AAA-ATPases) [5,7]. Of special interest, ABC transport systems that have been extensively studied as mediators of MDR in various types of cancer [8].

**Figure 1.**
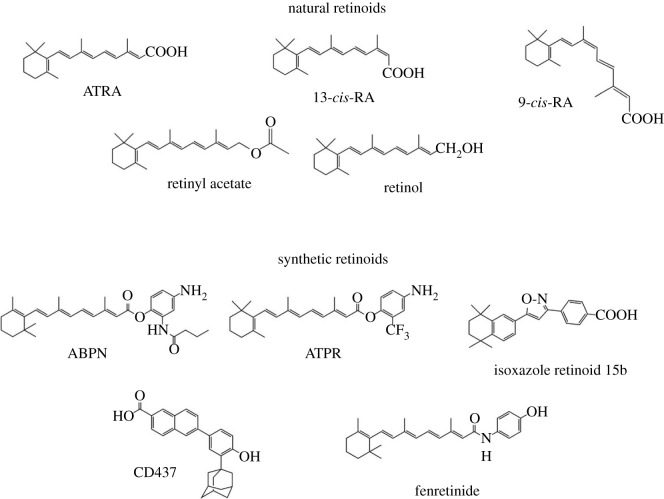
The chemical structures of multidrug resistance (MDR)-combating retinoids (natural and synthetic).

Although ABC transport systems are constitutively expressed in normal and cancer cells, their expression is also modulated by external factors, like retinoids. In the last few decades, it has become increasingly evident that retinoids, alone or in combinations, are promising anti-cancer compounds with considerable potency [9]. Significant correlation has been described between retinoids and ATPase transporters in cancer [10–12]. Besides the deregulation of ATPase gene expression after treatment with retinoids, ATRA and its analogues were found to be substrates for the MDR transporters and thus exposed to variations in their intracellular concentration leading to pharmacokinetic disturbances and variable anti-cancer response [13,14]. Figure 1 and table 1 summarizes the chemical structures and possible activity of retinoids by either re-sensitizing multiple MDR cell lines to chemotherapy or inducing direct growth inhibition of these MDR cells. Interestingly, the concentrations of retinoids needed for the demise of 50% of cultured cells growth (IC_50_) were found to be at, or slightly lower than, the micromolar scale [9,66,73,74]. Given this potency, more deep insights into the interplay between retinoids and MDR-conferring ATPases are still needed.

**Table 1.** The chemo-sensitizing effect of natural and synthetic retinoids on MDR cell lines.

retinoid	class	resistant cancer cell line	cancer origin	resistance to chemotherapeutic agent(s)	IC_50_ (µM)	Ref.
ATRA	retinoic acid receptor (RAR) Pan-agonist	MDA-MB-231	breast	paclitaxel (PTX) and 5-fluorouracil (5-FU)	34.1^b,c^	[15,16]
		LoVo/MDR	colon	doxorubicin (Dox)	NA^d^	[17]
		HEN-16-2/CDDP	cervical	cisplatin (CDDP)	NA^d^	[18]
		L1210/VCR	mouse lymphocytic leukaemia	vincristine (VCR)	NA^d^	[19,20]
		mS-0.5	melanoma	colchicine	NA^d^	[21]
		J82-NVB	bladder	navelbine	NA^d^	[22]
		HL60/DNR	acute promyelocytic leukaemia (APL)	daunorubicin	NA^d^	[23]
retinol	—	SW620	colorectal	etoposide	NA^d^	[24]
isoxazole retinoid 15b	RAR pan-agonist	HL60R	APL	ATRA	1.4^b,c^	[25]
		K562	leukaemia	ATRA	2.3^b,c^	[25]
		HUT78	T-cell lymphoma	ATRA	0.8^b,c^	[25]
fenretinide or 4-HPR	RAR-β selective agonist	Bel-7402	HCC	Dox and VCR	13.1^a^	[26,27]
		MDA-MB-231	breast	PTX and 5-FU	6.5^b,c^	[28]
		CHLA-119	neuroblastoma	ABT-737 (small-molecule BH3- mimetic) alone	NA^d^	[29]
ABPN (or CBG41)	RAR pan-agonist	MDA-MB-231	breast	PTX and 5-FU	3.3^b,c^	[28]
ATPR	RAR pan-agonist	MDA-MB-231	breast	PTX and 5-FU	18.1^b,c^	[15]

^a^IC_50_ (concentration of the compound caused 50% reduction in comparison to untreated cells) was calculated after 72 h of treatment.

^b^IC_50_ was calculated after 48 h of treatment.

^c^IC_50_ was calculated after 24 h of treatment.

^d^NA = not available.

## 2. The interplay between ABC transporters and MDR

Among the currently known ABC genes in the human genome, perturbations in the expression levels of some of these transporters are implicated in various human diseases, including cancer [30]. Furthermore, ABC transporters are highly associated with MDR [31–35]. Members of the ABCB, ABCC and ABCG subfamilies are major determinants for the emergence of MDR [36], Most importantly, P-gp, BCRP and multidrug resistance-associated protein-1 (MRP-1 or ABCC-1) are the best characterized [31–35]. The induction of the expression of these ATP-requiring proteins leads to significant changes in the signalling of many ions and molecules promoting tumorigenesis, including metal ions, vitamins and carbohydrates [5,37,38]. More profoundly, these efflux transporters pump a wide range of structurally diverse anti-cancer compounds outside the cell, reducing their bioavailability and therapeutic potential [1,39]. The overexpression of P-gp, BCRP and/or MRP-1 confers significant resistance to various neutral and cationic hydrophobic chemotherapeutic compounds [40–42]. These observations highlight the intimate link between disturbances in ABC transporters and conferred drug resistance, leading subsequently to increased tumour burden and reduced treatment outcomes.

As expected, inhibition of the pumping activity of the ABC transporter enzymes often leads to an increased cellular concentration of the cytotoxic drugs, and thus greater anti-cancer activity and reduced MDR [43]. Despite their structural differences, P-gp and BCRP share several common ligands that are transported across cell membrane [44–46]. These translocated ligands are collectively called allocrites [47]. Although they are functionally similar, P-gp and BCRP share only about 20% protein sequence identity in the NBDs with no significant sequence identity in the TMDs [48–51]. Nevertheless, both share various anti-cancer allocrites giving rise to MDR. The structural insights into the interactions between ABC transporters and their ligands show clearly that the hydrophobic nature of allocrites, including the retinoic acid and its analogues (or retinoids), is one of the major determinants of their ability to communicate with the transporters [48,52–58]. Finding chemosensitizers that are both effective and safe and could help rescue the emerging resistance to standard chemotherapeutic compounds in cancer is still needed.

## 3. Natural retinoids induce alternations in ABC transporters on different levels including expression, activity and binding interaction

The data available in the literature showed some examples of natural retinoids that proved their ability to modulate ABC transporters in cancer types on different levels and reverse MDR. For instance, retinol caused significant reduction in P-gp expression in colorectal carcinoma cells (CRC) leading to enhanced anti-tumor efficacy of etoposide [24,59]. In leukaemia, cell line L1210, ATRA caused transcriptional repression of P-gp enhancing the activity of verapamil substrate [19]. Interestingly, the latter effect was not attributed to the direct binding of RAR merely to the *ABCB1* promoter; instead, it appears to be mediated by RXR*α* sequestration after RAR-RXR*α* heterodimer formation [19]. As a result, fewer RXR*α* may be available to mediate the binding of ABCB1-activating progesterone-X-receptor (PXR) to *ABCB1* promoter. These results suggest that retinoic acid and related isomers attenuate the ABC transporter through modulation of mRNA expression levels.

On the level of ATPase activity, *Spodoptera frugiperda* (*Sf9*) membrane preparations expressing P-gp and BCRP was used to investigate the effects of some natural retinoids with verapamil and quercetin as their substrates respectively [10,11]. The study showed retinol and 13-*cis*-RA could significantly inhibit both the basal and the substrate-stimulated ATPase activity while ATRA, 9-*cis*-RA, retinyl-propionate and retinyl-palmitate did not have these effects. [11]. The ATPase inhibitory effect of retinoids observed in these experiments might be rooted in the hypothesis of retinoid-induced allosteric inhibition in activity of the transporters, related either to the competitive inhibition caused by direct interaction of retinoids with the substrate-binding site (s), or the membrane structural changes induced by retinoids.

On the level of binding interaction, studies revealed the interaction of ATPases with retinoic acid analogues to be stereospecific [11,53,60]. For example, 13-*cis*-RA inhibited both P-gp and BCRP transporters, while its stereoisomers ATRA and 9-*cis*-RA did not influence the enzymatic activity. Beside the stereo-selective binding of retinoids to P-gp and BCRP that occurs primarily at the level of the drug binding sites (allosteric sites) of the transporters, there is another level of binding at the plasma membrane itself from where the substrates and modulators probably interact with the drug binding site(s) [52]. The latter observation was confirmed by Fluorescence anisotropy assay using fluorescent membrane probe 1,6-diphenyl-1,3,5-hexatriene (DPH). Retinyl-acetate, 13-*cis*-RA, and retinol selectively increase the membrane viscosity and packing density in the depth of the membrane while, ATRA and 9-*cis*-RA did not have similar effects [11].

Calculating the kinetic parameters (*K*_m_ and *V*_max_) of the substrate-stimulated ATPase activity with or without retinoids showed retinol with higher *K*_m_ and lower *V*_max_ values of both transporters, suggesting mixed-type inhibition of P-gp and BCRP. Although 13-*cis*-RA showed mixed-type transporter inhibition of BCRP too, it caused a reduction of *V*_max_ with no significant increment of *K*_m_ value in the case of P-gp, emphasizing the non-competitive mode of inhibition of P-gp [11]. All these observations imply that natural retinoids with different stereoisomers have distinct modes of interaction and binding affinity with MDR-related ATPases, and suggest that minute differences in their structure might substantially influence the ATPase enzymatic activity.

## 4. The MDR-reversing activity of synthetic retinoids

The main obstacles of using formulations delivering natural retinoids into the systemic circulation are *in vitro* photo-instability [61–66] and *in vivo* enzymatic catabolism [67–70]. Therefore, there was urgent need for development of synthetic retinoic acid analogues, which mimic the biological actions and physico-chemical characteristics of natural ones, and to reverse MDR induced by cancer cells [9,71–76]. A heterocycle-containing retinoid called isoxazole retinoid 15b (figure 1) was synthesized and used to reverse the MDR activity of an acute promyelocytic leukaemia ATRA-resistant cell line called HL60R (table 1) [25]. This synthetic retinoid 15b rendered the cells more prone to the growth-inhibitory activity of ATRA and reactivated the cellular apoptosis pathway. Fenretinide [28], ABPN [28] and ATPR [15] (figure 1) are further examples of synthetic retinoids that were able to sensitize multi-drug resistant triple-negative breast cancer to paclitaxel and 5-fluorouracil. Nevertheless, these promising *in vitro* results need to be confirmed *in vivo* using chemo-resistant cancer animal models exposed to standard chemotherapeutics plus synthetic retinoids.

Despite this MDR-reversing activity in various cancers, an early report showed the cross-resistance to CD437 (a synthetic RARγ-selective agonist; figure 1) in paclitaxel-resistant human ovarian cancer cells which are overexpressing P-gp [77]. Others claimed that N-(4-hydroxyphenyl) retinamide (4HPR, aka fenretinide; figure 1) could potentiate the cytotoxicity of cisplatin in ovarian [78], breast [79] and lung [80] cancers. The underlying cause of this chemo-sensitization can be explained in light of the dose perspective point of view. Active doses from natural retinoids in blood are few in nanomolar range (1–20 nanomolar) [81] compared to the stable synthetic retinoids that can be taken through either parenteral or oral administration with relatively sufficient high local retinoid concentrations in the blood. The available doses of synthetic retinoids were able to subsequently block P-gp and BCRP expressed at the surface of resistant cancer cells [82–85].

## 5. Concluding remarks

Understanding of cancer resistance has evolved over the past few decades, and cancer resistance is suggested to be related to loss of retinoid-ABC transporter signalling. Also, emerging evidence sheds light on the development of MDR and the roles played by ATPases in chemotherapy resistance. Unfortunately, current chemotherapy regimens lead to limited efficacy and an upsurge in the number of cells with high levels of expression of ABC transporters. Various chemical compounds have been identified and tested to modulate or inhibit the transport function of ABC transporters, including P-gp and BCRP, and thus chemo-sensitize multidrug-resistant cancer cells. Nevertheless, ABC-modulatory compounds showing great potential on the bench frequently failed to prove efficiency in the clinic. Therefore, this presents a formidable challenge to medicinal chemists and structural biologists in defining P-gp and BCRP substrates with new structural diversity to modulate P-gp- and BCRP-mediated drug transport including retinoids. This requires precise knowledge of their structural domains and the exact mechanisms of interactions. Given that modulation of the ABC transporters might influence the pharmacokinetics of other co-administered chemotherapeutic drugs, more care should be taken upon the combination of retinoids with other anti-cancer drugs to avoid drug–drug interactions occurring at the level of the membrane transporters, P-gp and BCRP.

Considering the anti-cancer potency of synthetic retinoids, future research should focus on unravelling the impact of these compounds on the expression and activity of efflux pumps and other drug transporters. This could pave the way for recruiting synthetic retinoids as chemosensitizers that specifically target MDR-promoting transporters and could help in fighting the battle against chemoresistance.

## Data Availability

This article has no additional data.
